# Modulating Effect of Paeonol on Piglets With Ulcerative Colitis

**DOI:** 10.3389/fnut.2022.846684

**Published:** 2022-04-13

**Authors:** Shanshan Wang, Miaomiao Bai, Qingyan Shu, Zhengan Liu, Yirui Shao, Kang Xu, Xia Xiong, Hongnan Liu, Yao Li

**Affiliations:** ^1^Institute of Animal Nutrition, Northeast Agricultural University, Harbin, China; ^2^Hunan Provincial Key Laboratory of Animal Nutritional Physiology and Metabolic Process; National Engineering Laboratory for Pollution Control and Waste Utilization in Livestock and Poultry Production; Key Laboratory of Agro-ecological Processes in Subtropical Region; Hunan Provincial Engineering Research Center for Healthy Livestock and Poultry Production; Scientific Observing and Experimental Station of Animal Nutrition and Feed Science in South-Central, Ministry of Agriculture, Institute of Subtropical Agriculture, Chinese Academy of Sciences, Changsha, China; ^3^Key Laboratory of Plant Resources, Institute of Botany, Chinese Academy of Sciences (CAS), Beijing, China

**Keywords:** paeonol, ulcerative colitis, inflammatory factors, intestinal flora, intestinal barrier

## Abstract

Piglet enteritis is a major problem that needs to be solved urgently in modern pig production. Paeonol (Pae) has been used as a novel treatment option due to its good medicinal value. This study purported to elucidate the regulatory mechanism of Pae on dextran sodium sulfate (DSS)-induced ulcerative colitis (UC) in weaned piglets. A total of 36 crossbred (Duroc × Landrace × Yorkshire) weaned piglets were stochastically split into six groups: the control group, DSS group, 0.2% Pae group, 0.4% Pae group, 0.8% Pae group, and mesalazine group. The control and DSS groups were fed with a basic diet, the three Pae and mesalazine groups were fed with 0.2, 0.4, 0.8%, and 2 g mesalazine per kilogram of basic diet throughout the study. On the 15th day of the test period, the control group was gavaged with 10 ml of normal saline, while the remaining five groups were gavaged with 10 ml 5% DSS solution for 13 days. The study lasted for 27 days. The results showed that the 0.8% Pae group significantly increased the average daily feed intake (ADFI) and Occludin mRNA expression in the colon of piglets (*P* < 0.05). The 0.2% Pae group markedly increased the average daily gain (ADG) and zonula occludens-1 (ZO-1) mRNA expression (*P* < 0.05). In the 0.2% and 0.4% Pae groups, the feed-to-gain ratio (F/G) was significantly reduced and the mRNA expression levels of Caspase-8, respectively, markedly enhanced the mRNA expression levels of transforming growth factor-β (TGF-β) and interleukins-4 (IL-4) (*P* < 0.05). In the 0.8% Pae group, the relative abundance of *Campilobacterota* was significantly reduced (*P* < 0.05). In the 0.4% Pae group, the relative abundance of *Firmicutes* was notably increased (*P* < 0.05). In the 0.2 and 0.8% Pae groups, the relative abundance of *Prevotella* was markedly increased (*P* < 0.05). In the 0.2% Pae group, the contents of propionic acid, butyric acid, and valerate acid were markedly higher (*P* < 0.05). Thus, it is speculated that Pae may regulate the balance of anti-inflammatory/pro-inflammatory factors, improve intestinal tight junction expression, reduce apoptosis, and improve intestinal microflora structure and growth performance of piglets, thereby restoring intestinal barrier function and alleviating DSS-induced UC in piglets.

## Introduction

Inflammatory bowel disease (IBD), as a familiar multifactorial disease in modern large-scale pig farming, is one of the most serious piglet diseases (1). It is a multifactorial chronic gastrointestinal disease that causes abdominal pain, diarrhea, weight loss, malnutrition, and other conditions, including ulcerative colitis (UC) and Crohn's disease (CD) (1, 2). UC is a nonspecific inflammation of the gut that is characterized by persistent, diffuse, age- and gender-independent. Various complicated pathogenesis, such as immune factors, infection factors, apoptosis, genetic factors, and environmental factors, are all crucial for the onset of UC (3). UC is more prevalent than CD worldwide, especially in people aged 30–40. The prevalence and incidence are higher in North America, Asia, Africa, and Western Europe (4, 5). The main clinical manifestations of UC are diarrhea, bloody stools and bloody purulent stool. Severe patients are accompanied by high fever and poisoning symptoms (6). The lesion site of UC is mainly the colonic mucosa. The impaired function of the intestinal mucosal defense system and the resulting intestinal microorganisms disturbance are important mechanisms of UC (7, 8). The lesions of UC usually start from the rectum and extend proximally to part or the entire colon (9). Histologically, the destruction of epithelial cells, crypt abscesses, and breakages can be observed, with the presence of lymphocyte and neutrophil infiltration in the mucosa (10). Due to the complex causes of UC, multiple complications, and the risk of cancer (such as colorectal cancer), the treatment of UC is difficult (11, 12). Currently, the commonly used drugs for UC treatment are 5-aminosalicylic acid (5-ASA) (e.g., mesalazine and olsalazine), steroids (e.g., prednisone and thiopurines), and immunosuppressants (13). Different drugs should be used for the treatment depending on the severity of UC (14). For conditions that cannot be improved by drug treatment, partial rectum and colon are usually removed (15). However, long-term accumulation of these drugs can result in hepatorenal toxicity and injury, pancreatitis, weakened body resistance, and other adverse reactions (16). Therefore, it is necessary to develop a new treatment method for UC that has few side effects.

China is the hometown of traditional Chinese medicine (TCM). TCM has been widely used for thousands of years in the Chinese medicine and breeding industry and plays an important role. As medicine and feed additives, TCM has received increasing attention (17). Cortex Moutan (CM) is a dried root bark of *Paeonia suffruticosa*, namely, “Mudanpi” in Chinese, which has been directly used to treat various diseases in TCM, such as cardiovascular diseases and female reproductive diseases (18, 19). Paeonol (Pae), 2′-hydroxy-4′-methoxyacetophenone, is the main biologically active component of CM; has anti-inflammatory, antioxidant, antitumor, antidiabetic, neuroprotection, cardiovascular protection, and other pharmacological effects; and has been widely used in the clinical treatment of various diseases (20). It has been proved that Pae has significant effects on fever, headache, rheumatoid arthritis, coronary sclerosis, skin disease, and so on (21). However, due to the low bioavailability of Pae, different concentrations of Pae are prepared to address this problem, including tablets, gel, hydrogel, microparticles, and nanocapsules (22).

To study the pathogenesis and treatment options of UC, researchers usually establish various animal models to conduct experiments. These animal models provide the pathogenesis, pathological changes, and intestinal histological morphology of IBD and have become a common technique for studying IBD. They are usually classified into the model of spontaneous colitis, the chemical-induced colitis model, the transgenic animal model, and the adoptive transfer model (23), among which chemical-induced colitis is commonly used in animal experiments. Dextran sodium sulfate (DSS) is a water-soluble sulfate polysaccharide, which is useful for establishing UC models (24–26). The toxicity of DSS acts on colon epithelial cells to disrupt epithelial cell integrity, thereby causing damage and increasing the permeability of the colonic mucosa (27). As the DSS-induced UC model has the advantages of simple operation, wide application range, and symptoms that are very similar to human UC, it is widely used and proved to be matured by numerous trials (28, 29). In addition, the existing studies also have concluded that Pae can significantly increase colon injury, gradually restore crypt epithelial cells, reduce inflammatory cell infiltration, and improve the expression of related cytokines, proving that Pae can effectively improve the body's UC. These studies provide an important theoretical basis for our experiments (30–32). Currently, rodents are used in most animal UC models (such as rats and rabbits). In recent years, UC models in piglets have been gradually studied, but the mechanism of Pae on UC in weaned piglets has not yet been reported. Therefore, the objective of this experiment was to explore the mechanism and influences of Pae in the weaned piglets model of UC.

## Materials and Methods

### Materials

#### Material

Pae was purchased from Shanghai Maclin Biochemical Co. Ltd. (CAS: 552-41-0; ≥99%, Shanghai, China). DSS was purchased from Shanghai Aladdin Biochemical Technology Co. Ltd. (CAS: 9011-18-1; M.W 40000, Shanghai, China). Mesalazine enteric-coated tablets were provided by the Jiamusi Luling Pharmaceutical Co. Ltd. (H19980148, 0.25 g/tablet, Jiamusi, China).

#### Animals

Weaned piglets aged 30 days (Duroc × Landrace × Yorkshire) were purchased from Jiahe Agriculture stockbreeding Co. Ltd. (Hunan, China).

### Animal Experiment Design

**Figure 1:**
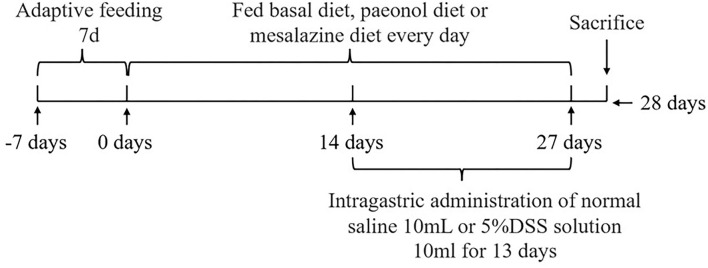
The experiment design.

The experimental scheme was approved by the Animal Welfare Committee of the Institute of Subtropical Agriculture, Chinese Academy of Sciences (2020-11A). A total of 36 healthy (Duroc × Landrace × Yorkshire) weaned piglets (30 days, 8 ± 0.5 kg) were selected and randomly divided into 6 groups, namely, the control, DSS, 0.2% Pae, 0.4% Pae, 0.8% Pae, and mesalazine groups. Each group consists of six replicates, with one piglet for each replicate. The control and DSS groups were fed the basal diet, while the three Pae and mesalazine groups were supplemented with 0.2, 0.4, and 0.8% Pae diets and 2 g mesalazine per kg basal diet, respectively. All piglets were fed the above diets during the whole experiment period, and the piglets were challenged with DSS from the 15th day. The control group received 10 ml of normal saline by gavage once a day, and the other five groups received 10 ml of 5% DSS solution by gavage once a day for 13 days. The experiment lasted for 27 days (Figure 1). The piggery should be thoroughly cleaned and disinfected before the experiment. Each piglet was raised in a single pen with *ad libitum* access to food and water daily. The piggery was kept well ventilated, the pen and foreign objects were cleaned on time, and the status and diarrhea degree of piglets were monitored and recorded every day. The corn-soybean basal diet was prepared according to the NRC (2012) standard. The specific feed ingredients are shown in Table 1.

**Table 1:** Basal diet composition and nutritional level of piglets.

**Ingredient**	**Content, %**	**Nutrient levels (calculated value)**	
Corn	54.00	Digestible energy (DE, Mcal/kg)	3.53
Defatted rice bran	4.50	Crude protein, %	17.33
Whey powder	3.00	Ca, %	0.80
Soybean meal	15.50	Total phosphorus (TP), %	0.63
Soybean oil	5.00	Available phosphorous (AP), %	0.36
Fish meal	6.30	Lysine, %	1.22
Extrude corn	8.07	Methionine, %	0.45
Calcium hydrogen phosphate	0.60	Threonine, %	0.73
Limestone	0.70	Tryptophan, %	0.47
Sodium chloride	0.60		
Zinc oxide	0.20		
Lysine	0.30		
Methionine	0.15		
Threonine	0.08		
Premix^a^	1.00		
Total	100.00		

a *Each kilogram of diet contains Zn (ZnSO_4_), 40.6 mg; Cu (CuSO_4_), 76 mg; Mn (MnSO_4_), 14.2 mg; Fe (FeSO_4_), 63.3 mg; I (Ca(IO_3_)_2_), 5 mg; Se (Na_2_SeO_3_), 4 mg; vitamin A, 8,255 IU; vitamin D3, 2,000 IU; vitamin E, 40 IU; vitamin B1, 2 mg; vitamin B2, 4 mg; pantothenic acid, 15 mg; vitamin B6, 10 mg; vitamin B12, 0.05 mg; nicotinic acid, 30 mg; folic acid, 2 mg; vitamin K3, 1.5 mg; biotin, 0.2 mg; choline chloride, 800 mg; vitamin C, 100 mg*.

### Growth Performance

The feed intake of each piglet was recorded every day, and their weight was recorded on the 1st, 14th, and 27th days of the experiment. Average daily gain (ADG), average daily feed intake (ADFI), and feed-to-gain ratio (F/G) were calculated.

### Disease Activity Index

The clinical symptoms of piglets, such as stool traits, weight change, and bloody stools, were observed. The severity of colitis was assessed using the disease activity index (DAI) every day, considering weight loss, stool consistency, and rectal bleeding (33). The occult blood in feces was evaluated using the benzidine method (Shanghai yuanye Bio-Technology Co. Ltd., Shanghai, China). The DAI scoring criteria were as follows: for weight loss, 0, no obvious damage to body weight; 1, 1–5% loss; 2, 6–10% loss; 3, 10–15% loss; 4, weight loss >15%; for stool consistency: 0, no diarrhea; 1, loose stools; 3, liquid stools; for rectal bleeding: 0, no bleeding; 1, occult bleeding; 3, gross bleeding.


$$\begin{array}{l} \text{DAI}=\frac{\text{weight loss score}+\text{stool consistency score}+\text{bleeding score}}{3}\; \end{array}$$

### Colon Weight/Length Ratio

At the end of the experiment, the piglets were slaughtered, and the colons without cecum were excised and rinsed with normal saline. The colon length was measured with a vernier caliper and weighed. The weight/length ratio is an indicator of colitis severity, and it is an indicator of colon weight gain caused by edema or chronic inflammation shortening (32).

### Microscopic Damage Score of Colon

After dissection, the distal colon of piglets was removed (about 1–2 cm) and fixed in 4% paraformaldehyde solution, followed by progressively increasing dehydration with 75 → 85 → 95 → 100 → 100% II ethanol, paraffin embedding and sectioning, and H&E staining, to assess the degree of inflammation. The colon specimens were detected using Biological Microscope (Eclipse Ci-L, Nikon, Japan) to characterize histopathological changes. The leading criteria were listed as follows (34): 0, natural colon tissue; 1, mucosa with mild inflammation or focal ulceration; 2, focal or extensive ulceration and inflammation of the mucosa or submucosa; 3, focal or extensive ulcerations and inflammation in the lamina propria; 4, serous membrane with focal or extensive ulceration and inflammation; and 5, serous membrane with extensive ulceration and transmural inflammation.

### Macroscopic Injury Score of Colon

Studies have reported a specific scoring system for assessing colon injury using the macro-inflammatory score (35), which is as follows: 0, no damage; 1, hyperemia but no ulceration; 2, linear ulcer with obvious inflammation; 3, linear ulcer with inflammation; 4, two or more sites of ulceration; 5, two or more major ulcers or inflammatory sites, one ulcer or inflammatory extending >1 cm along the length of the colon; 6–10, injury covered >2 cm along the length of the colon, the score increased by 1 for each additional centimeter of involvement.

### Serum Cytokine Levels

At the end of the experiment, blood was collected from the anterior vena cava of the piglets and centrifuged at 3,000 r/min for 5 min, and the upper serum was collected and frozen for storage. Using ELISA kit (MAISHA INDUSTRIES, Jiangsu, China), serum interleukin-1β (IL-1β), interleukin-6 (IL-6), interleukin-10 (IL-10), tumor necrosis factor-α (TNF-α), interferon-γ (IFN-γ), and immunoglobulin G (IgG) contents were detected.

### Intestinal-Microbiota 16sDNA Sequencing

Feces were collected from the colon at slaughter and stored in liquid nitrogen to detect gut microbes. The Magnetic Soil and Stool DNA Kit (Tiangen Biotech Co. Ltd, Beijing, China) was used to extract DNA from colonic contents. Primers 341F 5′- CCTAYGGGRBGCASCAG−3′ and 806R 5′- GGACTACNNGGGTATCTAAT−3′ containing specific bands were used to augment the V3–V4 region of RNA, and a 420 bp amplified fragment was obtained. The library was constructed using TruSeq DNA PCR-Free Library Preparation Kit (Illumina, USA) and sequenced using the Illumina NovaSeq platform (36).

### Real-Time Fluorescence Quantitative Detection of the Colon

The RNA from colon tissue was extracted using a Trizol kit (Invitrogen, Carlsbad, Canada) as described previously (37). After detecting the RNA concentration (ng/μl) using NanoDrop Spectrophotometer (NanoDrop, Wilmington, Del., USA), the RNA was reversed into cDNA based on the instructions of the TaKara reverse transcription kit SYBR Green qPCR Master Mix. The following steps were carried out in the PCR reaction program: 95°C pre-denaturation for 30 s, one cycle; 95°C denaturation for 5 s, 60°C annealing for 30 s, 40 cycles. A 20 μl reaction system was used for fluorescence quantification. The primer sequences of this study were provided by Sangon Biotech (Shanghai, China), as shown in Table 2.

**Table 2:** Gene and primer sequences.

**Gene**	**Accession No**.	**Primer 5′−3′**	**Size (bp)**	**Tm**°**C**
IL-4	NM_214123.1	F:TCACCTCCCAACTGATCCCA	144	60.18
		R:GCTCCATGCACGAGTTCTTT		
IFN-γ	NM_213948.1	F:TTCAGCTTTGCGTGACTTTG	121	57.53
		R:GGTCCACCATTAGGTACATCTG		
TGF-β	NM_214015.2	F:AAGCGGCAACCAAATCTATG	113	58.88
		R:CCCGAGAGAGCAATACAGGT		
ZO-1	XM_021098896.1	F:AGCCCCTCTTGATGCCTTTA	138	58.86
		R:CTCCATTGCTGTGTTCTCAAGT		
Occludin	NM_001163647.2	F:CAGGTGCACCCTCCAGATTG	111	60.68
		R:TGGACTTTCAAGAGGCCTGG		
Caspase-3	NM_214131.1	F:AGACGGACAGTGGGACTGAA	102	60.47
		R:GCCAGGAATAGTAACCAGGTG		
Caspase-8	NM_001031779.2	F:CCAGGATTTGCCTCCGGTTA	130	60.04
		R:CAGGCTCAGGAACTTGAGGG		
P65	NM_001114281.1	F:GGCACCGGATTGAGGAGAAA	87	60.25
		R:GGTCGGTGGGTCCATTGAAA		
TLR4	NM_001113039.1	F:CCGTCATTAGTGCGTCAGTTCT	100	60.67
		R:TTGCAGCCCACAAAAAGCA		
NF-κB	NM_001048232.1	F:TGGCTTCCCCCACTATGGAT	100	60.33
		R:GGTGTCTACAGTTCCGTGCT		

*IL-4, interleukins-4; IFN-γ, interferon-γ; TGF-β, transforming growth factor-β; ZO-1, zonula occludens-1; TLR4, toll-like receptor 4; NF-κB, nuclear factor kappa-B*.

### Western Blotting Analysis

The protein expression of P65, INF-γ, caspase-3, and intercellular cell adhesion molecule-1 (ICAM-1) in colon tissue was detected using Western blotting, as described by Shen et al. (38). Colon tissue was cut into small pieces and 10 times the volume of protein lysate was added, then phenylmethylsulfonyl fluoride (PMSF, 1:100) and phosphatase inhibitor were added, then shaken well, placed on ice, and lysed overnight at 4°C. The following day, the supernatant was centrifuged at 8,000 g (rpm) at 4°C for 10 min, and the protein concentration was determined using the BCA Protein Assay Kit (Servicebio, China). According to the concentration and volume, buffer solution was added to heat denaturation (95°C, 10 min), and SDS-polyacrylamide gel electrophoresis was performed to transfer the membrane and seal. The primary antibody was incubated at 4°C overnight, then washed with Tris-buffered saline Tween (TBST) and added to the fluorescent secondary antibody. Bio-Rad ChemiDoc XRS+ gel imaging system (Bio-Rad, USA) was used for development and gray scale analysis. Antibodies were used as follows: P65 (1:2,000; AF06781, Rabbit, AiFang biological, China); INF-γ (1:4,000, AF301097, Rabbit, AiFang biological, China); caspase-3 (1:2,000, AF06649, Rabbit, AiFang biological, China); ICAM-1 (1:2,000, AF6088, Rabbit, AiFang biological, China); and GAPDH (1:10000, YM3029, Mouse, ImmunoWay Biotechnology Company, USA).

### Statistical Analysis

All data were presented as mean ± SEM (*n* = 6). The Fisher's least significant difference *t*-test (LSD-t) multi-comparison method of PROC MIXED in the SAS9.4 software (SAS Institute Inc., Cary, NC, USA) was used for data analysis. The GraphPad Prism 8.0 software (GraphPad Software Inc., San Diego, CA, USA) was used to draw the histogram.

## Results

### Growth Performance

**Figure 2:**
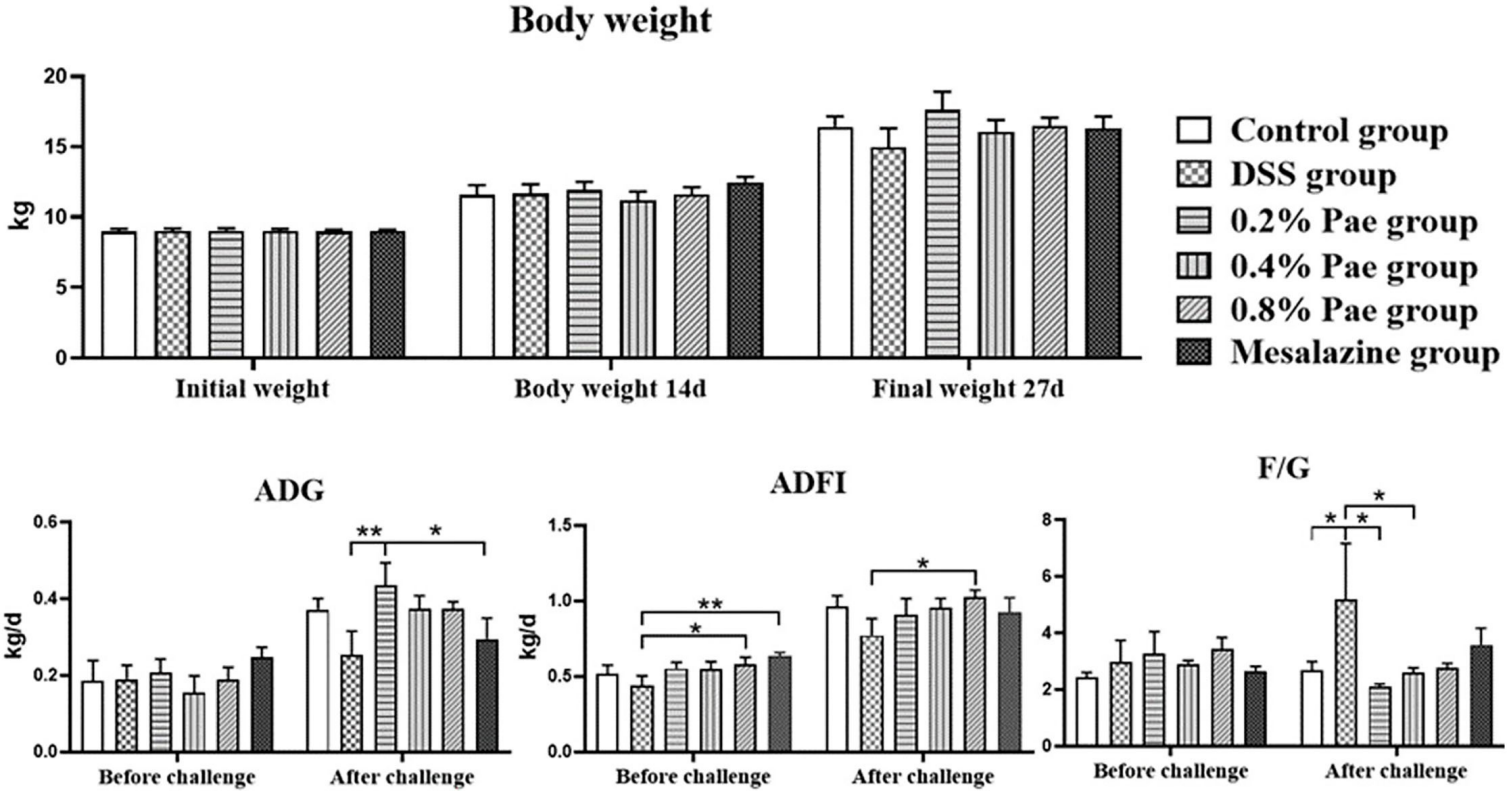
Effects of dietary supplementation of paeonol (Pae) on growth performance of UC piglets. Results are expressed as means ± SEM (*n* = 6). *indicated significant difference (*P* < 0.05), and **indicated extremely significant difference (*P* < 0.01). ADG, average daily gain; ADFI, average daily feed intake; F/G, feed-to-gain ratio.

As shown in Figure 2, the weight of piglets increased during the whole experiment, but there were no significant differences between the groups (*P* > 0.05). Compared with the DSS group, the ADFI in the 0.8% Pae group was significantly increased before and after the DSS challenge (*P* < 0.05). Compared with the DSS and mesalazine groups, the ADG in the 0.2% Pae group was significantly increased after the DSS challenge (*P* < 0.05). Compared with the control group, the F/G of piglets in the DSS group were significantly increased after the DSS challenge, while in the 0.2% and 0.4% Pae groups the increase in F/G caused by the DSS challenge was markedly reduced (*P* < 0.05).

### DAI Score and Colon Weight/Length Ratio

**Figure 3:**
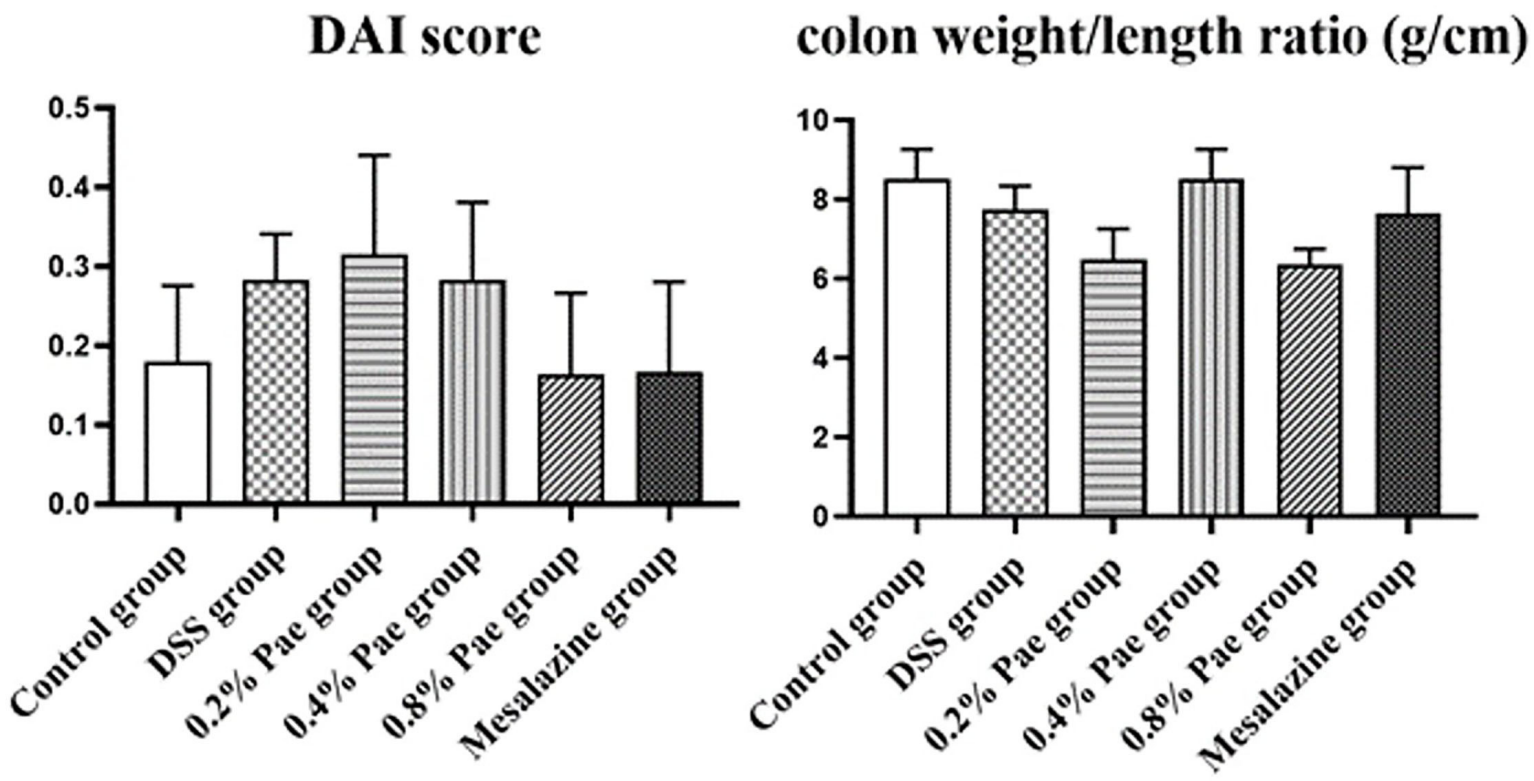
Effects of dietary supplementation of Pae on the DAI score and colon weight/length ratio of UC piglets. Results are expressed as means ± SEM (*n* = 6).

As shown in Figure 3, compared with the DSS group, the DAI scores of the 0.8% Pae and mesalazine groups, respectively, decreased by 42.86 and 39.29%, but there were no significant changes (*P* > 0.05). The colon weight/length ratio of the DSS group was 9.04% lower than that in the control group, but there was no significant change (*P* > 0.05), while the colon weight/length ratio of the 0.4% Pae group was restored to the same level as the control group. It showed that DSS caused damage to the colon, and Pae treatment alleviated the severity of UC to a certain extent.

### Macroscopic Damage Score and Microscopic Damage Score of Colon

**Figure 4:**
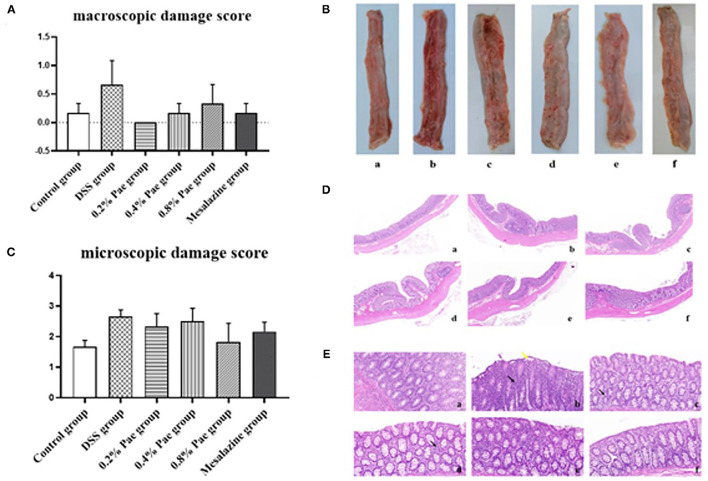
Effects of dietary supplementation of Pae on the colon macroscopic damage score **(A,B)** and the microscopic damage score **(C–E)** of UC piglets: (a) control group, (b) DSS group, (c) 0.2% Pae group, (d) 0.4% Pae group, (e) 0.8% Pae group, and (f) mesalazine group. **(D)** H&E staining, magnification: ×10, scale bar: 1,000 μm; **(E)** H&E staining, magnification: ×200, scale bar: 50 μm). Results are expressed as means ± SEM (*n* = 6).

As shown in Figure 4A, compared with the control group, the colon macroscopic damage score of DSS stimulation increased by 74.63%. Compared with the DSS group, the scores of the three Pae and mesalazine groups, respectively, decreased by 100, 74.63, 50.75, and 74.63%, but the differences were not significant (*P* > 0.05). As shown in Figure 4B, the DSS group had observable bleeding in the colon compared with the control group, while the three Pae and mesalazine groups could alleviate and recover the bleeding in the colon to some extent in a dose-dependent manner, suggesting that Pae could relieve DSS-induced UC.

As shown in Figure 4C, the DSS group had the highest histological score, and mucosal layer erosion was observed in the lamella propria, with a small number of epithelial cells locally exfoliated (Figure 4E, yellow arrow), and distinct diffuse lymphocytic infiltration in the lama propria (Figure 4E, black arrow). The histological scores of the 0.2, 0.4, and 0.8% Pae and mesalazine groups were slightly lower than those of the DSS group, but there were no significant changes (*P* > 0.05), and the lamina propria lymphocyte infiltration gradually decreased with increasing dose, and colonic epithelial structure was relatively complete (Figure 4D). At the same time, the colon tissue structure of the control group was complete, and the morphology and structure of epithelial cells were normal (Figure 4D).

### Serum Immunoglobulin and Cytokines

**Figure 5:**
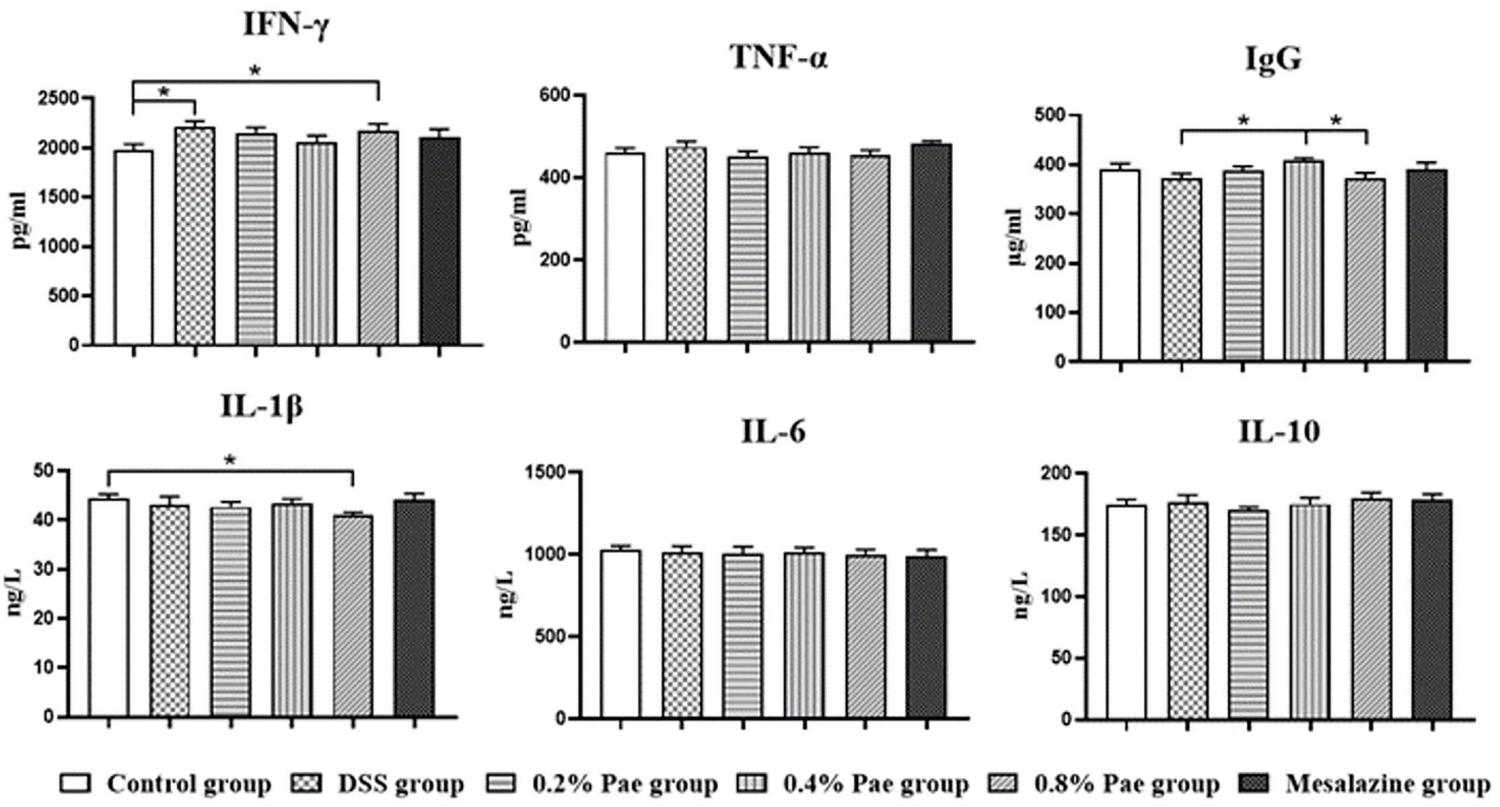
Effects of dietary supplementation of Pae on serum immunoglobulin and cytokines of UC piglets. Results are expressed as means ± SEM (*n* = 6). *indicated significant difference (*P* < 0.05). IFN-γ, interferon-γ; TNF-α, tumor necrosis factor-α; IgG, immunoglobulin G; IL-1β, interleukins-1β; IL-6, interleukins-6; IL-10, interleukins-10.

As shown in Figure 5, the serum IFN-γ content of the DSS and the 0.8% Pae groups was markedly increased (*P* < 0.05). Compared with the DSS and 0.8% Pae groups, the serum IgG content of the 0.4% Pae group was significantly increased (*P* < 0.05). Compared with the control group, the serum IL-1β content of the 0.8% Pae group was significantly decreased (*P* < 0.05). These results suggest that dietary Pae supplementation can improve immune function to a certain extent.

### Relative Cytokine MRNA Levels in the Colon

**Figure 6:**
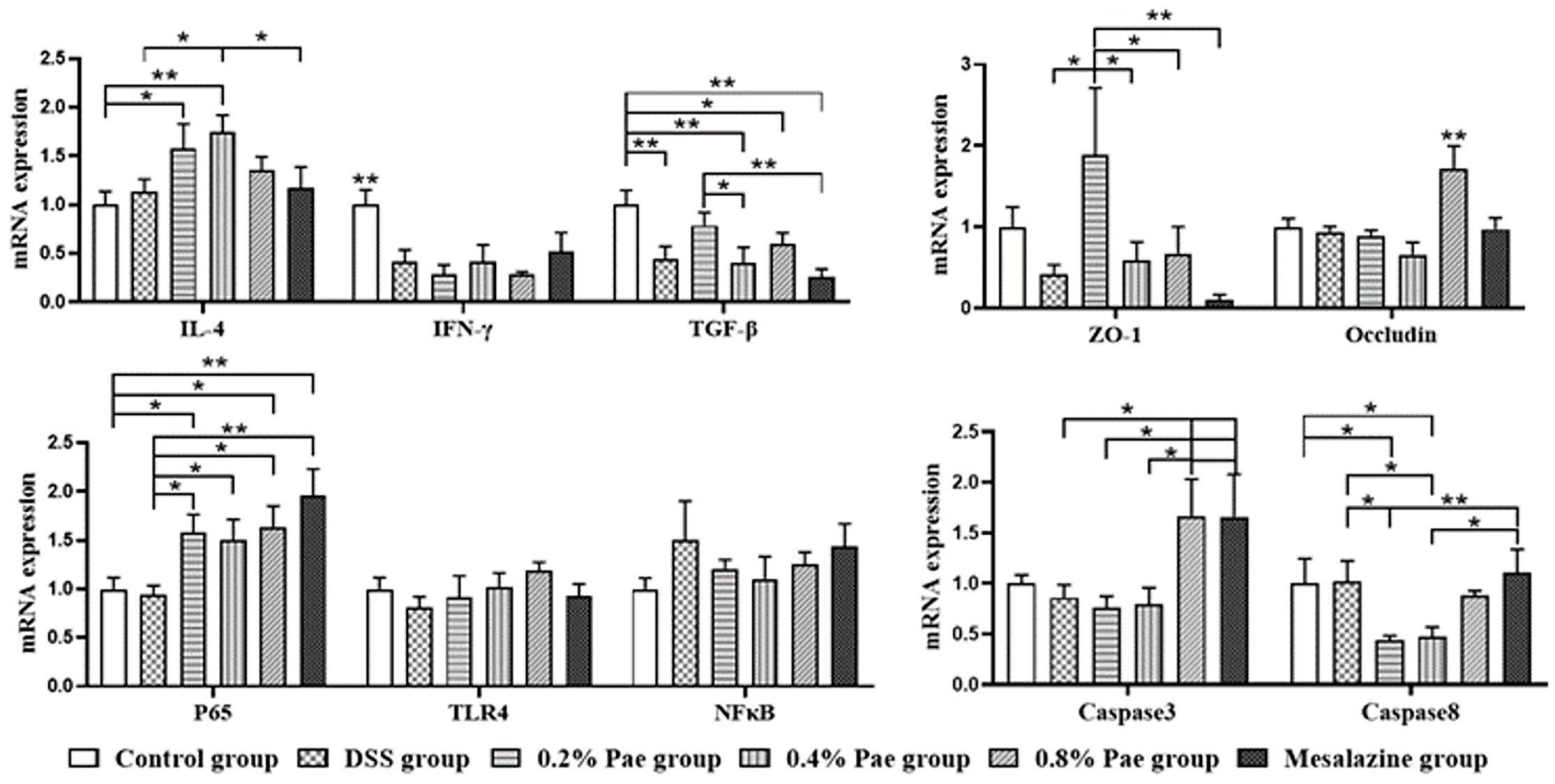
Effects of dietary supplementation of Pae on the expression of genes related to cytokines, tight junctions, cell apoptosis, and NF-κB signaling pathway in UC piglets colon. Results are expressed as means ± SEM (*n* = 6). *indicated significant difference (*P* < 0.05), and **indicated extremely significant difference (*P* < 0.01). IL-4, interleukins-4; IFN-γ, interferon-γ; TGF-β, transforming growth factor-β; ZO-1, zonula occludens-1; TLR4, toll-like receptor 4; NF-κB, nuclear factor kappa-B.

As shown in Figure 6, in terms of cytokines, compared with the control and DSS groups, the IL-4 mRNA expression (*P* < 0.05) of the 0.2% and 0.4% Pae groups was significantly increased. Compared with the control group, the other five groups all showed a significant decrease in the IFN-γ mRNA expression level (*P* < 0.05). Compared with the control group, TGF-β mRNA expression levels in DSS group, 0.4% Pae group and mesalazine group were significantly decreased (*P* < 0.05). Compared with 0.4% Pae group and mesalazine group, 0.2% Pae group significantly increased TGF-β mRNA expression (*P* < 0.05). In terms of tight junctions, the ZO-1 mRNA expression in the colon of the 0.2% Pae group was significantly increased, and the Occludin mRNA expression level (*P* < 0.05) of the 0.8% Pae group was significantly increased. In terms of cell apoptosis, the expression of caspase-3 mRNA of the 0.8% Pae and mesalazine groups was significantly increased, while the relative expression of caspase-8 (*P* < 0.05) of the 0.2% and 0.4% Pae groups were markedly decreased. In the NF-κB signaling pathway, compared with the control and DSS groups, the P65 mRNA expression was significantly increased in the three Pae and mesalazine groups (*P* < 0.05) but had no significant influence on mRNA expression of TLR4 and NF-κB (*P* > 0.05).

### Expression Levels of Cytokines in the Colon

**Figure 7:**
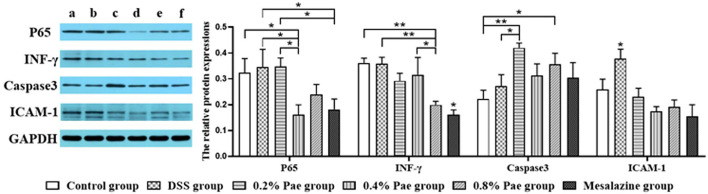
Effects of dietary supplementation of Pae on the expression of related factor proteins in UC piglets colon: (a) control group, (b) DSS group, (c) 0.2% Pae group, (d) 0.4% Pae group, (e) 0.8% Pae group, and (f) mesalazine group. Results are expressed as means ± SEM (*n* = 6). *indicated significant difference (*P* < 0.05), and **indicated extremely significant difference (*P* < 0.01). IFN-γ, interferon-γ; ICAM-1, intercellular cell adhesion molecule-1.

As shown in Figure 7, the P65 protein expression in the 0.4% Pae and mesalazine groups was notably reduced compared with the DSS and 0.2% Pae groups (*P* < 0.05). The protein expression of INF-γ was markedly decreased in the 0.8% Pae group compared with the control, DSS, and 0.4% Pae groups, while the mesalazine group was significantly decreased compared with all other groups (*P* < 0.05). The protein expression of Caspase3 in 0.2% Pae group significantly increased compared with the DSS group (*P* < 0.05). Compared with the control group, the protein expression of caspase-3 was markedly increased in both the 0.2% and 0.8% Pae groups (*P* < 0.05). Compared with the DSS group, the protein expression of ICAM-1 in the other five groups was significantly decreased (*P* < 0.05).

### Colonic Microflora of UC Piglets

#### Alpha Diversity and Operational Taxonomic Units Abundance of Colonic Microflora

**Figure 8:**
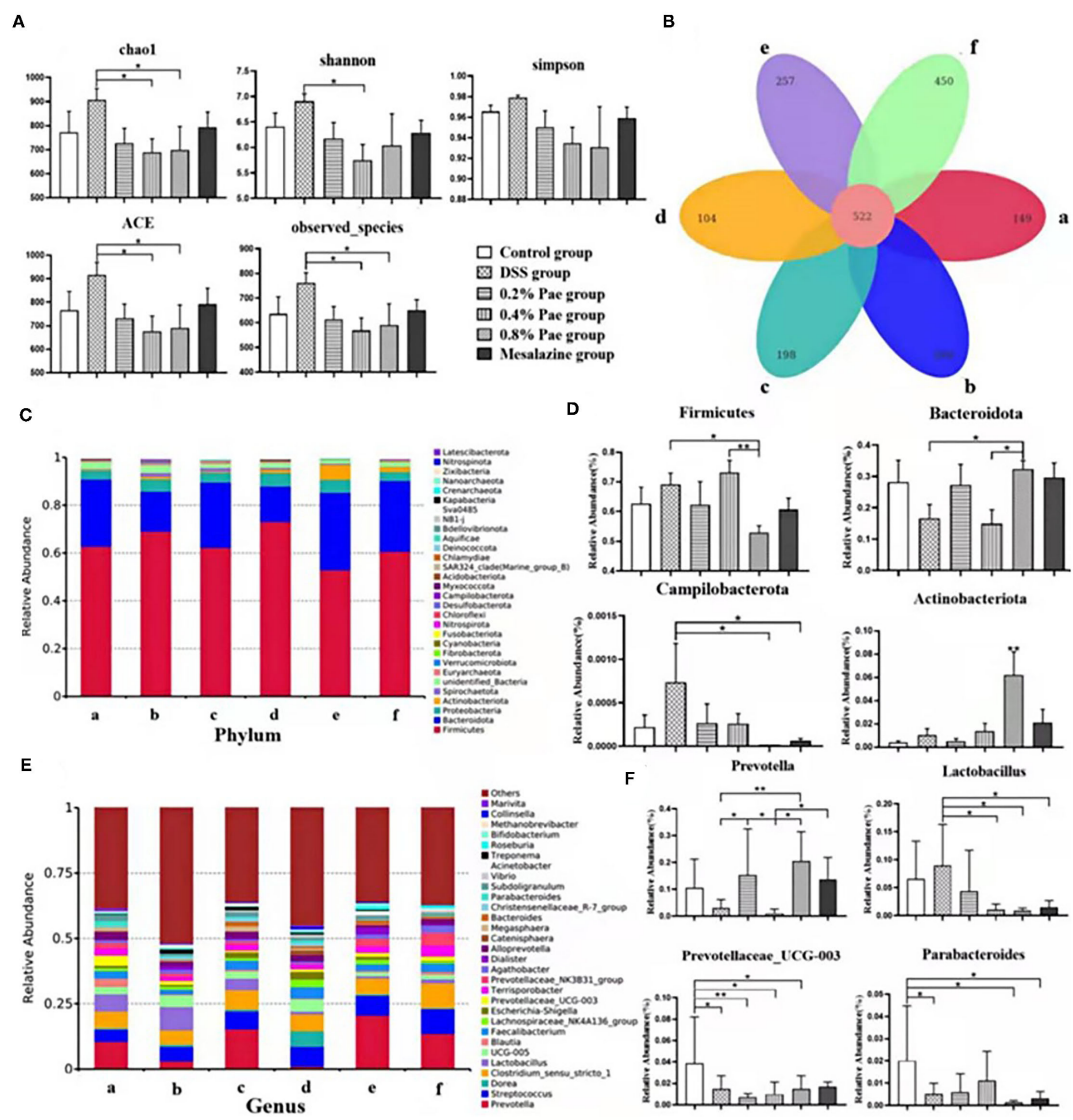
Effects of dietary supplementation of Pae on colonic microflora alpha diversity and Venn graph **(A,B)**, relative abundance at the phylum level **(C,D)**, and relative abundance at the genus level **(E,F)** of UC piglets: (a) control group, (b) DSS group, (c) 0.2% Pae group, (d) 0.4% Pae group, (e) 0.8% Pae group, and (f) mesalazine group. Results are expressed as means ± SEM (*n* = 6). *indicated significant difference (*P* < 0.05), and **indicated extremely significant difference (*P* < 0.01).

As shown in Figures 8A,B, compared with the DSS group, species abundance, chao 1, and ACE indices were significantly decreased in the 0.4 and 0.8% Pae groups, and the Shannon index was significantly decreased in the 0.4% Pae group. The total number of OTUs in the six groups is 522, accounting for 26.91% of the total. The number of OTUs unique to the control, the DSS, the three Pae, and the mesalazine groups are 149, 260, 198, 104, 257, and 450, respectively.

#### Colon Microflora Composition

We analyzed phyla with an average abundance >0.1% of colonic microflora of UC piglets at the phylum level (Figures 8C,D). The main colonic microbes of UC piglets in the six groups were Firmicutes, Actinobacteriota, Bacteroidota, Proteobacteria, and Spirochaetota. Among them, Firmicutes, Bacteroidota, and Proteobacteria are the dominant flora in the colon content (Figure 8C). Compared with the DSS and 0.4% Pae groups, the relative abundance of Bacteroidota in the 0.8% Pae group was significantly increased (*P* < 0.05), and the relative abundance of Actinobacteriota in the 0.8% Pae group was also significantly increased compared with the other five groups (*P* < 0.05). Compared with the 0.8% Pae group, the relative abundance of Firmicutes (*P* < 0.05) in the 0.4% Pae group was notably increased. Compared with the DSS group, the relative abundance of Campilobacterota (*P* < 0.05) in the 0.8% Pae and mesalazine groups was notably decreased.

At the genus level (Figures 8E,F), the main dominant flora in the colonic flora of UC piglets includes *Prevotella, Streptococcus, Lactobacillus, Dorea, Clostridium, Blautia, Faecalibacterium, Lachnospiraceae_NK4A136_group, Prevotellaceae_UCG-003*, and *Escherichia-Shigella*. Compared with the DSS group, the relative abundance of *Prevotella* (*P* < 0.05) in the 0.2% and 0.8% Pae groups was markedly increased. The *Lactobacillus* abundance decreased notably in the 0.4% Pae, 0.8% Pae, and mesalazine groups (*P* < 0.05). Compared with the control group, the relative abundance of *Parabacteroides* in the DSS, 0.8% Pae, and mesalazine groups was notably decreased (*P* < 0.05). The abundance of *Prevotellaceae_UCG-003* in the DSS and three Pae groups was also dramatically decreased compared with the control group (*P* < 0.05).

### Short-Chain Fatty Acids in Colon Contents

**Figure 9:**
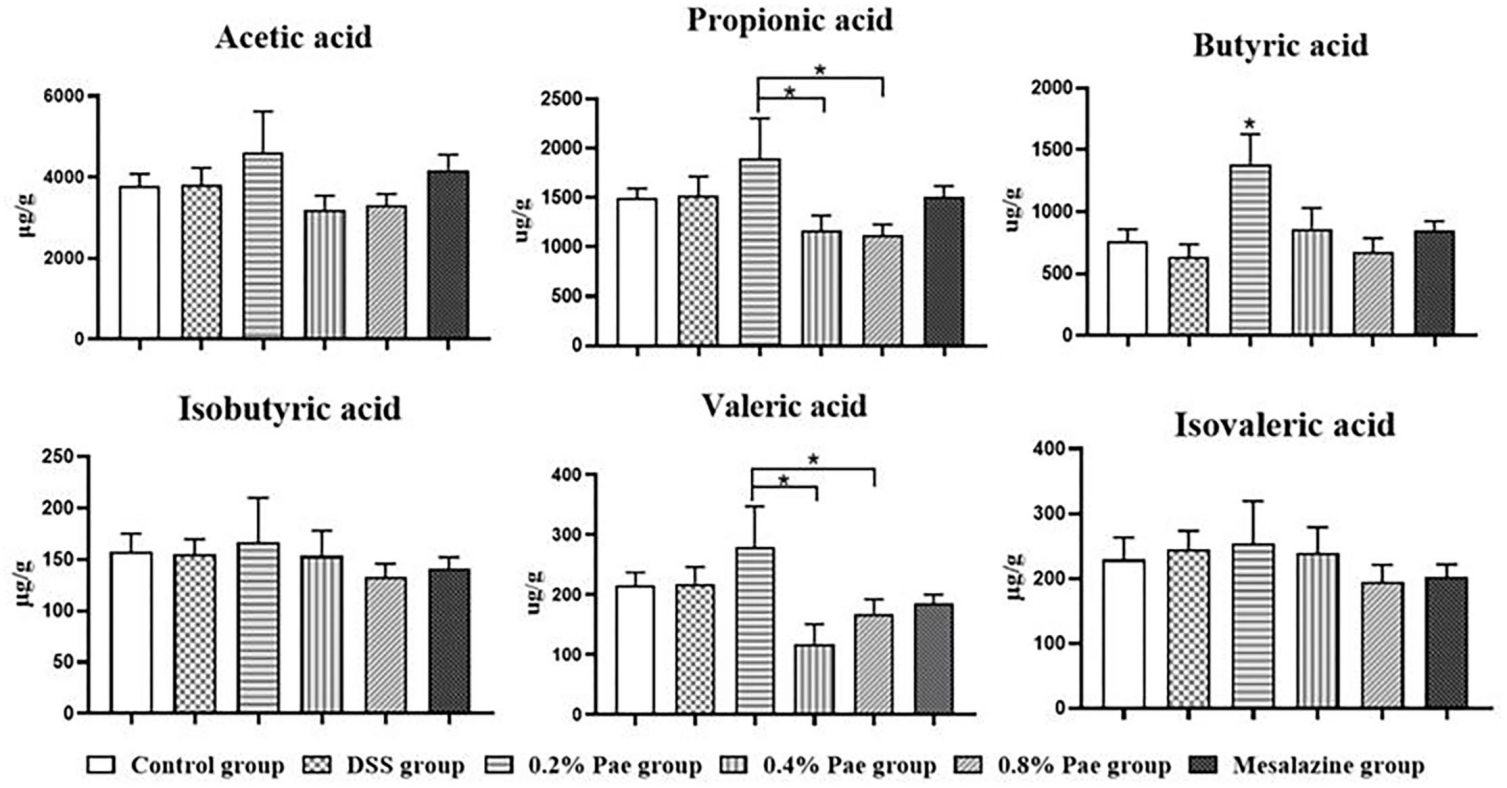
Effects of dietary supplementation of Pae on SCFAs in colonic contents of UC piglets. Results are expressed as means ± SEM (*n* = 6). *indicated significant difference (*P* < 0.05).

As shown in Figure 9, the contents of propionic acid and valeric acid in colonic contents in the 0.2% Pae group was significantly increased compared with the 0.4% and 0.8% Pae groups (*P* < 0.05), and the butyric acid content was significantly increased compared with the other five groups (*P* < 0.05).

## Discussion

Ulcerative colitis is a chronic, nonspecific bowel disease characterized by ulcerative lesions of the colonic mucosa that can involve the entire colon (39). When UC happens, the organism causes some conditions such as diarrhea and rectal bleeding, making the patient's weight drop substantially and leading to death (40, 41). The intractability of UC seriously endangers the health and life quality of patients and brings indelible losses to patients. Currently, the efficacy of the existing treatment methods for UC is limited, so it is necessary to find new methods to treat UC. Studies have shown that the DSS challenge can cause severe diarrhea in piglets, reduce their weight, and reduce the gain to feed ratio (42, 43). The objective of this study was to investigate the effects of Pae on DSS-induced UC piglets. In this study, the weight of piglets showed an upward trend throughout the experiment. Dietary supplemented with 0.2 and 0.8% Pae significantly increased the ADG and ADFI of the piglets, respectively. After the DSS challenge, the F/G ratio of the piglets increased significantly, while 0.2 and 0.4% Pae supplementation significantly improved this trend. These results indicate that DSS does damage the growth performance of piglets, and the addition of Pae to the diet can ameliorate the growth performance of UC piglets, which indicates that dietary supplementation of Pae has certain positive roles on the growth performance of piglets. But so far, studies on Pae as a feed additive to promote the growth and development of animals have not been reported, and Pae mostly uses drugs as targets for disease research. Therefore, we speculated that Pae could improve the growth performance of piglets by exerting anti-inflammatory and antioxidant effects, improving the immune function and alleviating the loss caused by DSS induced in piglets. Therefore, we will discuss the anti-inflammatory and antioxidant effects of Pae on UC.

As early as 1985, DSS has been used to induce UC, and more and more studies have adopted the DSS model to study the pathogenesis and influencing factors of UC (44). We can also induce different degrees and locations of UC by controlling the molecular weight, concentration, duration, and frequency of DSS (25, 45). It has been proved by research that DSS challenge can damage the colon, especially the colonic mucosa, destroy the intestinal barrier, cause diarrhea or hematochezia, and reduce body weight, resulting in a significant increase in the DAI score and the colon weight/length ratio. Histologically, UC is characterized by epithelial cell destruction, mucin depletion, crypt abscess, and infiltration of neutrophils and lymphocytes (25), which are all typical features of UC. Kim's study found that after DSS-induced UC, piglets showed severe diarrhea and blood in feces, histologically observed crypt structure distortion, and inflammatory cells infiltrated the mucosa and submucosa (43). Zong and Kazuhiro have proved that Pae can alleviate the body's UC by reducing the DAI score, the colon weight/length ratio, the macroscopic damage score, and the microscopic damage score (31, 32). In this study, the same results were found. DSS challenge caused colon injury, while the three Pae supplementation groups could reduce the DAI score, the colon weight/length ratio, relieve colon bleeding, restore colonic lamina proper, and reduce lymphocyte infiltration to some extent. These results reveal that Pae has certain therapeutic and ameliorative effects on UC.

Interferon-γ is a pivotal cytokine in the pathogenesis of IBD, so most studies have chosen it as a therapeutic target (46). In this study, the DSS challenge significantly enhanced the content of pro-inflammatory factor IFN-γ in serum, suggesting that the DSS model was successful to a certain extent. In addition, the expression of IFN-γ protein in the colon was significantly decreased in the 0.8% Pae group. IgG is the most abundant immunoglobulin in human serum, and it is of great significance in antigen binding, formation of immune complexes, and activation of specific cells (47). Studies have proved that UC is closely related to IgG, and IgG antibody as a therapeutic target of UC has been widely studied (48–50). Studies on the effect of Pae on IgG have not been reported, but the results of this study showed the 0.4% Pae group significantly increased the IgG content. These results suggest that Pae may improve immune function and relieve UC. In the DSS-induced UC model, TGF-β levels were significantly decreased, while the IFN-γ, IL-1β, and IL-6 levels were increased (51). Pae can regulate inflammation and oxidative stress, and Ge et al. proved that Pae can increase the levels of TGF-β1 and IL-10, and decrease the levels of IL-1β, IL-6, IL-8, and TNF-α, and can regulate intestinal immune disorders, thereby improving colitis (30). We found that 0.2% and 0.4% Pae groups significantly increased the TGF-β and IL-4 mRNA expression levels, respectively, which were consistent with previous studies. ICAM-1, a member of the immunoglobulins superfamily (IGSF) of adhesion molecules, participates in the inflammatory cascade and plays a special role in the pathogenesis of IBD (52). Previous studies have proved that ICAM-1 is closely related to neutrophils, and the changes of neutrophils can be observed in the pathogenesis of UC, so ICAM-1 can also be used as a therapeutic target for UC (52, 53). In this study, DSS significantly increased the protein expression of ICAM-1 in the colon, while the three Pae and mesalazine groups decreased the expression of ICAM-1. These results indicate that adding Pae to the diet can improve the expression of inflammatory factors and improve the immune function of the body to a certain extent, showing that Pae has a certain alleviating effect on UC.

The expression of tight junction complexes and the integrity of epithelial cells are strongly linked to the intestinal barrier, so changes in these two factors usually mean impaired intestinal barrier function (25). Some studies have shown that during the occurrence of UC, the tight junction complex of the intestine changes first, which increases the permeability of the intestine, and the accumulation of inflammatory factors leads to further inflammation (54–56). Our study found that the 0.2 and 0.8% Pae groups significantly increased the ZO-1 and Occludin mRNA expression levels in the colon, respectively, indicating that Pae can improve intestinal tight junction expression and restore intestinal mucosa. It has been proved that UC induces apoptosis of epithelial cells by producing IL-13 by T cells, leading to further pathological changes of UC (57). Apoptosis and necrosis can cause the death of intestinal epithelial cells, aggravate the inflammatory response, and further exacerbate the severity of UC (58). Caspase-8 is an exogenous proapoptotic caspase that mediates RIPK3 and activates inflammasome (59). The mRNA expression level of caspase-8 was notably decreased in 0.2 and 0.4% Pae groups. Therefore, we speculate that Pae may alleviate UC in piglets by enhancing intestinal tight junctions expression, reducing cell apoptosis, and maintaining intestinal mucosal recovery.

The NF-κB signaling pathway is one of the important ways to regulate the body's inflammatory response and is involved in the transcription and regulation of some genes. Activation of IκB kinase, degradation of IκB protein, and nuclear translocation of P65 are the main pathways of NF-κB signal transduction (60). The nuclear transport of P65 is the key to the function of NF-κB (61). A large number of studies have proved that NF-κB can promote the expression of pro-inflammatory factors, such as TNF-α and IL-1β, and is considered to be closely related to the occurrence of UC (62). Studies have shown that in UC patients, NF-κB family members were significantly expressed, and IκB was significantly phosphorylated and degraded, while Pae can inhibit the expression of NF-κB (63, 64). These results showed that the 0.4% Pae group significantly decreased the protein expression of P65, the three Pae groups significantly increased the mRNA expression of P65, and there were no significant differences in other NF-κB-related genes. Our results are slightly different from previous results, so we speculate that Pae may play a role in relieving UC through other signaling pathways by regulating the expression of P65, but the specific regulatory mechanism needs further study.

Under normal circumstances, intestinal epithelial cells, immune cells, and intestinal microbiota interact to achieve a balanced state. The intestinal flora and its products are crucial in the pathogenesis of IBD (56). When inflammation occurs, the intestinal microbiota becomes unbalanced, which further exacerbates inflammation (65). Studies have shown that the number of Enterobacteriaceae and *Clostridium* spp. increased after DSS induced colitis, the Bacteroidetes and Firmicutes abundance decreased, and the Proteobacteria and Actinobacteria abundance increased (66, 67). We observed that the 0.4 and 0.8% Pae groups reduced the species abundance, chao 1, and ACE indices in the alpha diversity of the flora. In addition, the relative abundance of Bacteroidota and Actinobacteriota in the 0.8% Pae group was significantly increased, while the abundance of Campilobacterota was significantly descended. The 0.4% Pae group dramatically advanced the relative abundance of Firmicutes. The relative abundance of *Lactobacillus* was also decreased in the 0.4 and 0.8% Pae groups. The 0.2 and 0.8% Pae groups enhanced the relative abundance of *Prevotella*. Intestinal flora can not only maintain intestinal homeostasis and affect immune function but also secrete metabolites such as SCFAs, which play a pivotal role in maintaining body health (68). SCFAs can maintain the body's energy metabolism homeostasis, provide energy for intestinal cells, protect the intestinal barrier, and participate in the body's immune regulation (69). Among them, acetic acid and propionic acid can be produced by *Bacteroides*, while butyric acid is produced by Firmicutes (70). We found that among the three Pae supplementation groups, the 0.2% Pae group notably increased the content of propionic acid, butyric acid, and valeric acid in SCFAs. Therefore, we speculate that Pae may improve the intestinal barrier and relieve UC by improving the structure of the intestinal flora and the content of SCFAs.

In our experiment, different doses of Pae were set up to study the relieving effect of DSS-induced UC. The results obtained in this experiment were combined with actual pig production, showing that dietary supplemented with 0.2% Pae played the best role in alleviating UC. Therefore, 0.2% Pae is recommended as the optimal supplemental level to improve UC in piglets.

## Conclusion

This study showed that DSS-induced UC in piglets caused colonic damage and intestinal barrier destruction, leading to inflammatory reactions in piglets, which seriously affected the health of piglets. Effects of dietary supplementation of Pae regulate the balance of anti-inflammatory/pro-inflammatory cytokines, improve the structure of the intestinal flora, restore colon tissue structure and intestinal barrier function, and promote the growth performance of piglets, thus alleviating the severity of UC in piglets. Considering the actual demand for pig production, we suggest that 0.2% Pae should be selected as the optimal supplemental level to improve the UC of piglets. This study provides evidence for Pae in the treatment of UC in piglets and also suggests that Pae has potential medicinal value in the treatment of IBD and is expected to become a new therapeutic strategy. However, whether Pae is beneficial for human IBD remains questionable, so further in-depth studies are needed.

## Data Availability Statement

The original contributions presented in the study are publicly available. This data can be found at: https://www.ncbi.nlm.nih.gov/search/all/?term=PRJNA797584.

## Ethics Statement

The animal study was reviewed and approved by Animal Welfare Committee of the Institute of Subtropical Agriculture, Chinese Academy of Sciences.

## Author Contributions

YL and HL contributed to the conception and design of the study. SW conducted all experiments, analyzed the data, and wrote the first draft of the manuscript. HL, MB, YS, and YL contributed to manuscript revision, read, and approved the submitted version. HL provided financial support. QS, ZL, KX, and XX supervised. All authors contributed to the article and approved the submitted version.

## Funding

This project was supported by a grant from the National Natural Science Foundation of China (32072741), the Innovation Team in Key Area Innovation Team of Physiology and Metabolism and Body Health in Pig (2019RS3022), the Youth Innovation Promotion Association (CAS, 2019356), and the China Agriculture Research System (CARS-35).

## Conflict of Interest

The authors declare that the research was conducted in the absence of any commercial or financial relationships that could be construed as a potential conflict of interest.

## Publisher's Note

All claims expressed in this article are solely those of the authors and do not necessarily represent those of their affiliated organizations, or those of the publisher, the editors and the reviewers. Any product that may be evaluated in this article, or claim that may be made by its manufacturer, is not guaranteed or endorsed by the publisher.
